# Age differences in learning emerge from an insufficient representation of uncertainty in older adults

**DOI:** 10.1038/ncomms11609

**Published:** 2016-06-10

**Authors:** Matthew R. Nassar, Rasmus Bruckner, Joshua I. Gold, Shu-Chen Li, Hauke R. Heekeren, Ben Eppinger

**Affiliations:** 1 Department of Cognitive, Linguistic, and Psychological Sciences, Brown University, Providence, Rhode Island 02912, USA; 2 International Max Planck Research School LIFE, Max Planck Institute for Human Development, 14195 Berlin, Germany; 3 Department of Education and Psychology, Freie Universität Berlin, 14195 Berlin, Germany; 4 Department of Neuroscience, University of Pennsylvania, Philadelphia, Pennsylvania 19104, USA; 5 Department of Psychology, TU Dresden, 01069 Dresden, Germany

## Abstract

Healthy aging can lead to impairments in learning that affect many laboratory and real-life tasks. These tasks often involve the acquisition of dynamic contingencies, which requires adjusting the rate of learning to environmental statistics. For example, learning rate should increase when expectations are uncertain (uncertainty), outcomes are surprising (surprise) or contingencies are more likely to change (hazard rate). In this study, we combine computational modelling with an age-comparative behavioural study to test whether age-related learning deficits emerge from a failure to optimize learning according to the three factors mentioned above. Our results suggest that learning deficits observed in healthy older adults are driven by a diminished capacity to represent and use uncertainty to guide learning. These findings provide insight into age-related cognitive changes and demonstrate how learning deficits can emerge from a failure to accurately assess how much should be learned.

## Abstract (web summary)

The ability to learn decreases with old age especially in a dynamically changing environment, however the precise nature of this decline is not understood. Nassar and colleagues report that older adults show a reduced ability to learn from uncertain outcomes compared to younger adults.

## Graphical abstract

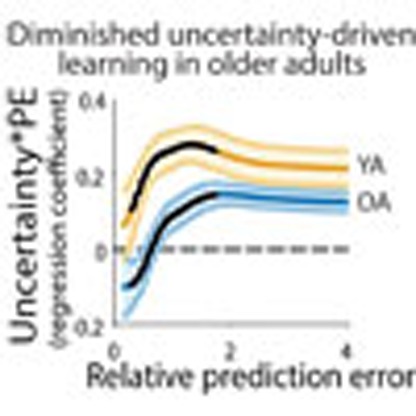

The ability to learn new outcome contingencies declines over the course of healthy aging in humans and animals^1^,^2^. One proposed mechanism is age-related deficits in the ability to compute prediction errors^3^. However, evidence for this hypothesis is mixed^1^,^2^,^4^,^5^. For example, learning ability and biomarkers of prediction error signalling are diminished for older versus younger adults in tasks that require learning probabilistic reward contingencies from experience^3^,^5^,^6^. However, no such differences are found for gambling tasks with pre-specified reward contingencies^7^,^8^,^9^,^10^,^11^,^12^. One possible explanation for these findings is that age-related deficits in error-driven learning do not reflect changes in how prediction errors are computed but rather how they are regulated according to environmental statistics^13^,^14^,^15^,^16^. Such regulation should, for instance, enhance learning when contingencies are unknown (in periods of high uncertainty) but, if anything, suppress learning when contingencies are stable and known (low uncertainty)^17^.

Our goal was to test whether and how age differences in factors that regulate error-driven learning can account for age-related deficits in adaptive behaviour^18^,^19^,^20^,^21^. We focused on three factors—uncertainty, surprise and hazard rate—that can have distinct effects on learning and are subserved, at least in part, by dissociable neural mechanisms^14^,^15^,^22^.

Uncertainty about the state of a latent variable, such as the underlying market value of a company whose stock price fluctuates wildly, is termed estimation uncertainty^19^,^23^. When estimation uncertainty is high, corresponding beliefs are unreliable, and should be revised according to new outcomes through learning^13^,^18^,^23^. More precise prescriptions for the rate of learning are provided by relative uncertainty, which describes the contribution of estimation uncertainty to overall ignorance about upcoming events (for example, tomorrow's stock price). Relative uncertainty sets the optimal learning rate in a stable environment; thus a stock trader should be more sensitive to new stock prices when she is less certain about the underlying value of the company.

While uncertainty can provide a reasonable prescription for learning during periods of relative stability, efficient learning in dynamic environments also requires online detection of abrupt shifts in a latent state, such as might occur for a company with the announcement of a costly settlement^24^. Such abrupt shifts are referred to as change points. They render the past irrelevant to the future, and thus require an immediate increase in learning to discard irrelevant information^13^,^17^. While change points cannot always be perfectly identified, the probability of such an event can be efficiently estimated according to Bayes rule^18^,^21^,^24^. In the Bayesian formulation, change-point probability depends critically on the likelihood of the newest observation (today's stock price) given the previous data (price history). The less likely the new observation, the more it indicates a fundamental change in the latent state. Thus a primary determinant of change-point probability, and consequently learning, is surprise^17^,^18^,^19^,^21^. Surprise is greatest when outcomes deviate most substantially from predictions and thus can be measured by the absolute magnitude of prediction errors^25^.

The probability that a surprising observation reflects a change point depends on the base rate of change points in the environment, termed the hazard rate^21^. The hazard rate acts as a prior that regularizes the number of detected change points; thus the lower the hazard rate, the greater the amount of surprise that is tolerated without an increase in learning. That is to say, a moderate decrement in the stock price of a company should be largely ignored if that company has been historically stable, but demands rapid recalibration of beliefs if that company has been prone to fundamental price shifts in the past.

Despite evidence that uncertainty, surprise and hazard rate are important mediators of learning in young adults, little is known about how subjective representations of these quantities affect learning across the adult lifespan. Older adults show deficits in learning tasks involving uncertainty and changes in task contingencies^6^,^26^,^27^,^28^,^29^,^30^,^31^,^32^. In principle, such learning deficits could reflect a specific computational deficit in any of the normative learning factors listed above, but this idea has not been tested explicitly.

Here we provide such a test, using a three-step analytic approach. First, we used a normative model to show that deficits in processing uncertainty, surprise or hazard rate can lead to unique and diagnosable learning deficits. Second, we collected and analysed behavioural data to identify signatures of these different impairments and found that older adults have a deficit in representing and using uncertainty in the service of learning. Third, we confirmed this finding through quantitative model fitting and identified an additional age-related difference: older adults tended to be more variable in their assignments of learning rate than younger subjects. Altogether, these findings support the idea that age-related differences in learning reflect a selective impairment in the ability to represent uncertainty rather than a general deficit in the ability to learn from prediction errors.

## Results

### Task design and model predictions

**Figure 1:**
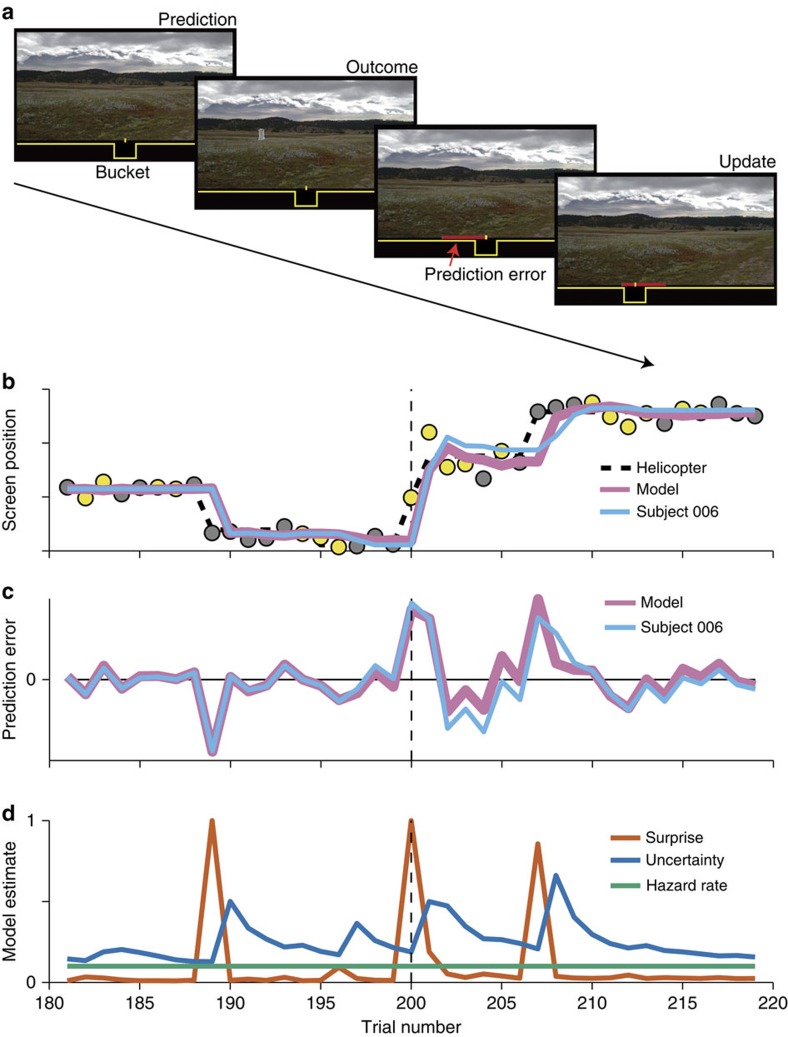
Predictive inference task. (**a**) Participants were instructed to place a bucket under the most likely location of a helicopter to collect gold coins (prediction). On each trial the helicopter dropped a bag and some of the contents from this bag fell in the bucket placed by the participant (outcome). After bag contents were revealed and distributed (not shown) tick marks were displayed to mark the bucket and bag locations from the previous trial along with a red line indicating the difference between these locations (prediction error). Participants then updated the position of the bucket to maximize earnings on the subsequent trial. (**b**) Screen positions (ordinate) of various task events are plotted across trials (abscissa) for part of a task session. On most trials the helicopter (black dotted line) remained in the same location as the previous trial, though occasionally it would move to a completely new location of the screen (change point). On each trial the helicopter would drop a bag containing either gold or rocks (yellow and grey points, respectively) that was offset from the helicopter position according to a noise function that was manipulated blockwise (vertical dotted line indicates block transition). The participant placed the bucket on each trial according to previous bag locations (blue line). Participant bucket placements were qualitatively consistent with a normative learning model (pink line). (**c**) Spatial prediction errors (bag location–bucket location) for the same example participant (blue) and model (pink) were relatively small during periods of stability but extreme at change points. (**d**) Normative learning requires information about hazard rate, surprise and uncertainty on each trial. Hazard rate (green) was constant for the entire task leading the model to rely on the surprise associated with each prediction error to estimate trial-to-trial variability in change-point probability (orange). Likely change points lead to elevated estimates of relative uncertainty (blue) that decay with each well-predicted observation.

Younger (20–30 years; *n*=57) and older (56–80 years; *n*=57) adults performed a modified version of a predictive inference task administered as part of a larger battery of behavioural tasks^15^. The task required participants to infer the position of a virtual helicopter based on the positions of bags that had previously fallen from it using a form of error-driven learning (Fig. 1a). On each trial, the participant placed a bucket under the inferred position of the helicopter. A bag then fell from the top of the screen and exploded to reveal contents that could be collected in the bucket. The participant could adjust the bucket placement for the subsequent trial in response to the indicated error between the previous bucket placement and the subsequent bag location. The key manipulation was the trial-by-trial bag position, which was normally distributed around a mean determined by the helicopter location. The mean location was stable for sequences of trials separated by abrupt, un-signalled change points at which the helicopter relocated to a random position. The s.d. of the bag distribution was consistent across change points within a block of trials but manipulated across blocks to give rise to varying levels of noise.

Effective performance in this task, as has been demonstrated by young adult participants in previous studies, is well described by a normative model that uses learning rates that are dynamically adjusted on each trial^15^,^16^,^18^,^21^,^24^ (Fig. 1b,c). Learning rate reflects the extent to which a given error is used to update the bucket position. A small learning rate (∼0) implies that an error should be ignored, whereas a large learning rate (∼1) implies that the error should be used in its entirety to place the bucket at the most recent bag location.

Dynamic learning rates prescribed by the normative model depend on two factors: change-point probability and relative uncertainty. The first factor, change-point probability, reflects the probability that the helicopter has relocated immediately before the most recent bag drop and can itself be dissociated into two separate components: hazard rate and surprise. The hazard rate reflects the frequency of change points, which is constant throughout the task but likely perceived differently across subjects (Fig. 1d, green). Surprise reflects the lack of correspondence between predictions and outcomes and varies from trial-to-trial according to the absolute magnitude of prediction errors (Fig. 1d; orange). Thus, trial-to-trial fluctuations in change-point probability can be thought of as a normative prescription for surprise-driven learning.

The second factor, relative uncertainty, reflects the imprecision with which helicopter position can be estimated based on previous bag locations. Relative uncertainty is greatest after the model has observed only a single bag drop from a new helicopter location and decreases with each additional (unsurprising) bag leading to learning rates that decay during periods of stability (Fig. 1d, blue).

**Figure 2:**
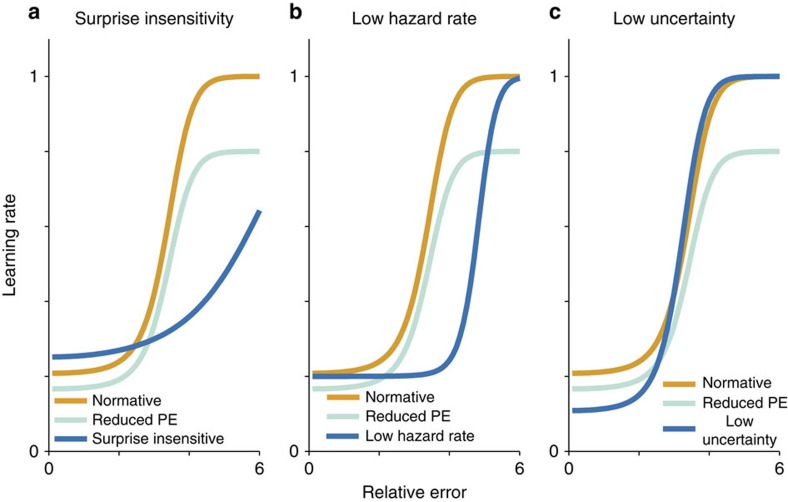
Learning deficits stemming from hypothetical deviations from normative behaviour. Normative learning (orange line, all panels) requires identifying and responding appropriately to surprising outcomes while maintaining internal representations of uncertainty. While deficits in learning could emerge from a failure to fully represent prediction errors (green line, all panels), they could also emerge from a more selective deficit in appropriately choosing a learning rate such as: (**a**) Insensitivity to surprising outcomes, (**b**) Underestimation of the true base rate of change points (hazard rate) or (**c**) Insufficient representation of uncertainty. Such deviations from normative behaviour can all reduce the rate of learning, but do so only under a specific set of conditions. Insensitivity to surprise should lead to learning deficits most notable after surprisingly large errors (**a**). Underestimation of the hazard rate should result in learning deficits for moderately surprising errors (**b**). Reduced uncertainty should lead to selective learning impairments for the least surprising outcomes (**c**).

One important feature of our model is that it can provide quantitative predictions of the distinct effects that specific computational impairments have on learning (Fig. 2). Insensitivity to surprise is characterized by a decrease in the steepness of the function relating relative prediction error to learning rate. Thus, surprise insensitivity would lead to a specific reduction in learning from large errors (Fig. 2a). In contrast, underestimation of the hazard rate increases the threshold for surprise necessary to infer a change point, without affecting the slope of the function. This leads to reduced learning rates across a wide range of moderately surprising outcomes that are less likely to be interpreted as change points if a low hazard rate is assumed. Underestimation of uncertainty leads to reduced learning specifically from small, unsurprising prediction errors that are unlikely to reflect change points in the helicopter location (Fig. 2c). All of these effects can also be distinguished from overall changes in learning rate, such as those expected from decreased prediction error magnitude (Fig. 2a–c, green).

### Age-related differences in learning

**Figure 3:**
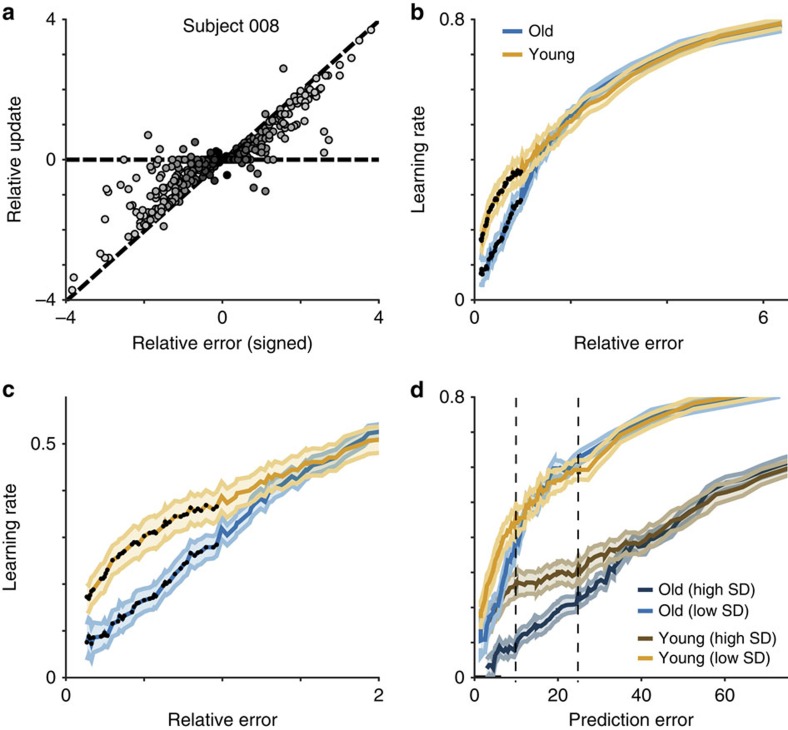
Younger participants adjusted expectations more after making relatively small errors. (**a**) Relative bucket updates (bucket update divided by noise; ordinate) are plotted against signed relative error (error magnitude divided by noise; abscissa) for each trial completed by a single example subject. Trials are divided into bins according to unsigned relative error (absolute error magnitude divided by noise; lighter colours for larger relative errors). Consistent with normative learning, lighter points tend to be closer to the unity line (dotted diagonal) and darker points tend to deviate towards the zero update line (dotted horizontal). (**b** and **c**) Regression coefficients describing the effect of prediction errors on updates are plotted separately for older (blue) and younger (orange) participants across all sliding window bins of unsigned relative error (**b**) and for a subset of smaller relative error bins (**c**). Significant differences (permutation tests for cluster mass, *n*=57 per group, *P*<0.05) are marked with dark points. (**d**) Differences in learning depended on the noise distribution. Learning rates predicted by the regression model for old (blue) and young (orange) participants are plotted separately for high (dark) and low (light) standard deviation conditions across bins of spatial error magnitude. Learning differences between groups emerged for both conditions for errors smaller than one standard deviation of the noise distribution (marked by dotted lines). In all panels, lines and shading reflect mean and s.e.m. respectively.

To test whether learning differences across healthy aging reflect one or more of these specific computational deficits, we first analysed participant behaviour using a regression model. The model described learning behaviour according to principles of error-driven learning. Specifically, the distance that participants moved the bucket on each trial (the update) was explained using the difference between the position of the bag and the previous bucket position, called the prediction error (Fig. 1a). The slope of such a linear function is equivalent to a fixed learning rate in an error-driven learning model. The regression model included separate interaction terms that could account for learning rates that were adjusted on each trial according to surprise, uncertainty and hazard rate (Fig. 3a). To diagnose the computational impairments highlighted above, we applied the regression model in sliding windows of trial outcomes ordered from the least to the most surprising as indexed by relative error magnitude (see Methods section).

Learning rates described by the regression model tended to increase with relative error magnitude, as predicted by the normative model, but with age-related differences for relatively small errors (Fig. 3b–d). On average, younger adults were more influenced than older adults by small errors (permutation test for H_0_: equal mean learning coefficients, *n*=57 per group, *P*<0.05). This reduced sensitivity to small errors in older adults did not reflect age-related differences in visual acuity. A deficit in visual acuity would have led to impairments in the processing of small errors independently of the noise level. However, we found that the age-related differences in behaviour were highly sensitive to expected variability, with pronounced differences between young and older participants for moderate spatial errors when the variability in the bag distribution was large (high noise; Fig. 3d).

**Figure 4:**
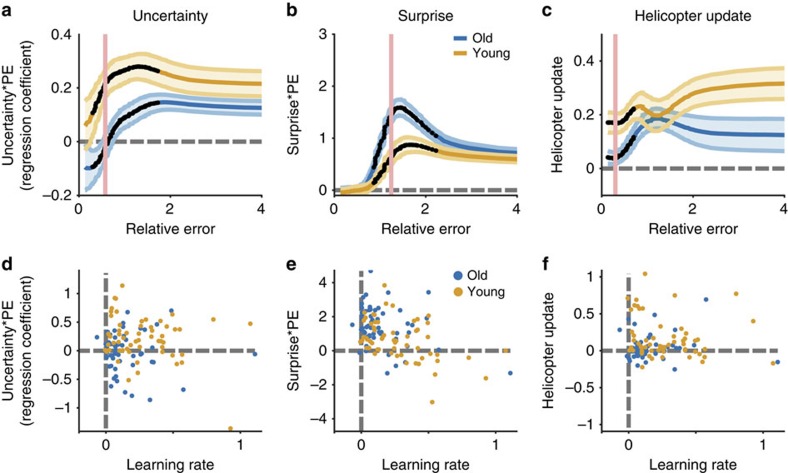
Age differences in uncertainty and surprise-driven learning. Regression coefficients for interaction terms were estimated in sliding windows of outcomes ordered from the least to the most surprising. For **a**–**c**, regression coefficients are plotted on the ordinate against the median binned relative error for each sliding window on the abscissa. (**a**) Uncertainty regression coefficients (ordinate) for older (blue) and younger (orange) participants differed across a range of relative errors (abscissa). (**b**) Surprise regression coefficients (ordinate) were larger in older, relative to younger, participants for moderately large errors (abscissa). (**c**) Helicopter update coefficients (ordinate) show that both groups moved the bucket towards the helicopter on catch trials (positive values), but that young participants did so more than old participants after making small relative errors (abscissa). Mean/s.e.m. coefficient values are reflected by line/shading and dark points indicate bins including a significant group difference (permutation tests for cluster mass, *n*=57 per group, *P*<0.05). (**d**–**f**) Regression coefficients for individual young/old participants (orange/blue points) in relative error bins with maximal group differences (pink lines in **a**–**c**; ordinate) are plotted against learning rate coefficients. Note that group coefficient differences are not driven by the small group of subjects with extremely high learning rates.

These differences in overall learning rate were accompanied by age-related differences in how specific computational factors governed learning. The normative model suggests that learning should be greater during periods of uncertainty or after observing a surprisingly large prediction error (Fig. 1d). Our regression model captured such behaviour with interaction terms reflecting the effects of uncertainty and surprise on prediction error-driven learning. In Fig. 4 we plot the coefficients for these interaction terms for each sliding window of data sorted from least to most surprising. Positive coefficient values for these interaction terms indicate higher learning rates on trials where trial-by-trial estimates of relative uncertainty (uncertainty) or change-point probability (surprise) were greater. Consistent with normative updating, we found that both age groups learned more when uncertainty was high, especially for intermediate relative prediction errors (Fig. 4a; permutation test for H_0_: uncertainty coefficient=0, *n*=57, *P*<0.001 for young participants and *P*<0.005 for older participants) albeit with substantial heterogeneity across individuals (Fig. 4d). Similarly, both groups increased learning as a function of surprise (Fig. 4b,e; permutation test for H_0_: surprise coefficient=0, *n*=57, *P*<0.001 for both groups). However, younger participants adjusted learning according to uncertainty more than their older counterparts (Fig. 4a,d; permutation test for H_0_: equal mean uncertainty coefficients, *n*=57 per group, *P*<0.005). In contrast, older participants adjusted learning more in response to surprise than their younger counterparts (Fig. 4b, permutation test for H_0_: equal mean surprise coefficients, *n*=57 per group, *P*<0.005). Thus, our data show an age-related double dissociation with respect to the factors that govern adaptive learning: older adults show a reduced sensitivity to uncertainty but an enhanced sensitivity to surprise than younger adults.

Behaviour on ‘catch trials' (trials on which the helicopter was visible) also showed age-related learning differences. Participants could use this information as an additional cue to identify the true mean of the helicopter location. Using additional terms in our regression model, we found that both younger and older participants tended to make appropriate, additional updates towards the visible helicopter (Fig. 4c,f; permutation test for H_0_: coefficient=0, *n*=57, *P*<0.001 for both groups) and away from the most recent bag position (Supplementary Fig. 3; permutation test for H_0_: coefficient=0, *n*=57, *P*<0.001 for both groups). However, the groups differed in the extent to which they updated towards the helicopter: older participants showed less pronounced updating, particularly after the smallest errors (Fig. 4c; permutation test for H_0_: equal mean helicopter update coefficients, *n*=57 per group, *P*<0.05).

These learning differences between groups were reflective of age rather than differences in fluid intelligence or working memory. An explanatory model that included each of the age-related learning differences identified above (learning rate for unsurprising outcomes, uncertainty, surprise and helicopter updating) could explain differences in age across subjects (*F*=6.37, *n*=114, *P*<0.001) and generated predictions that correlated with fluid intelligence, as assessed with Raven's progressive matrices (*r*=−0.31, *P*<0.001) and working memory as assessed by the operation span task (*r*=−0.35, *P*<0.001). However, learning differences were not simply reflecting aspects of fluid intelligence or working memory that co-vary with age, as the task measurements were related to age even after accounting for these covariates (nested *F*=3.29, *n*=114, *P*=0.01).

### Learning differences are simulated by reduced uncertainty

To qualitatively assess the contribution of uncertainty underestimation to age-related differences in task performance, we modified the normative model by artificially reducing its estimate of uncertainty on each trial. We used both the normative model and the low-uncertainty model to generate data for the task sessions completed by our human participants and analysed the data from these models using the same regression framework. Furthermore, we followed the same procedure to simulate task behaviour using two other sub-optimal models, one insensitive to surprise and the other with under-estimated hazard rates, to compare the simulated behaviour to our empirical results.

**Figure 5:**
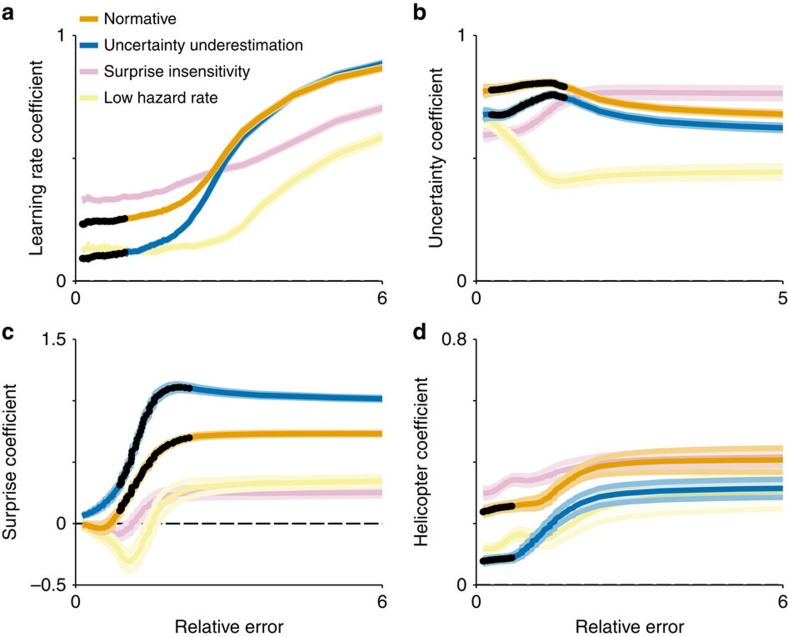
Uncertainty underestimation mimics altered behaviour in older participants. Behaviour was simulated using a normative model (orange) and three deviant models: (1) surprise insensitivity (pink), (2) hazard rate underestimation (yellow) and (3) uncertainty underestimation (blue). Colours were chosen to emphasize that uncertainty underestimation deviates from the normative model in the same manner that older participants deviate from younger ones. (**a**) Mean/s.e.m. learning rate regression coefficients (line/shading) describing the effect of the prediction errors on the subsequent updates made by these models (see Fig. 3b). Only the uncertainty underestimation model shows a selective learning deficit in these bins. (**b**) Mean/s.e.m. uncertainty regression coefficients (line/shading) are plotted separately for each simulated model across all relative error bins (compare with Fig. 4a) (**c**) Mean/s.e.m. surprise regression coefficients (line/shading) are plotted separately for each simulated model across relative error bins (compare with Fig. 4b). (**d**) Mean/s.e.m. regression coefficients (line/shading) describing the effects of the helicopter over simulated model updates for trials when it was visible (for example, how much does the model adjust estimates towards the observed helicopter; compare with Fig. 4c). In all panels, dark points mark bins with significant differences between younger and older participants (permutation tests for cluster mass, *n*=57 per group, all *P* values <0.05) and lines/shading reflect mean/s.e.m. of simulated model behaviour.

The low-uncertainty model, unlike the other sub-optimal models, generated predictions that differed from those of the normative model in a manner that mimicked four key differences between younger and older participants. First, the low-uncertainty model showed lower learning rates specifically after small errors (Fig. 5a). Second, the low-uncertainty model modulated learning less according to uncertainty (Fig. 5b). Third, the low-uncertainty model was more sensitive to changes in surprise, particularly after the moderately sized errors where older and younger participants differed most (Fig. 5c). Fourth, the low-uncertainty model was less prone to adjust expectations towards an additional cue representing the visible helicopter, especially in the absence of a surprisingly large prediction error (Fig. 5d). Neither of the other two sub-optimal models could reproduce the specific learning deficit for small errors or the double dissociation between uncertainty- and surprise-driven learning effects.

### Reduced uncertainty best describes learning in older adults

To quantitatively test the idea that learning differences across the age groups arise as a result of uncertainty underestimation, we fit a flexible version of the normative learning model directly to participant behaviour. This flexible model contained free parameters to describe each of the computational deficits that could impact learning rate: (1) insensitivity to surprise, (2) mis-estimated hazard rate and (3) uncertainty underestimation. In addition to these learning terms, the model also contained two parameters to allow for behavioural variability: one term that allowed for variability in update magnitude (allowing for imprecise bucket placements) and another that allowed for variability in learning rate (allowing for bucket precision to decrease with error magnitude). For each subject, all parameters were fit simultaneously using maximum likelihood estimation and best fitting parameters were interpreted as quantitative estimates of the latent factors governing learning behaviour. Note that parameter fits from this model should be viewed as complimentary to, but not independent from, the results from our descriptive analysis as the parameter fits provide an aggregate measure of computational deficits based on the same underlying data used for the regression analysis above.

**Figure 6:**
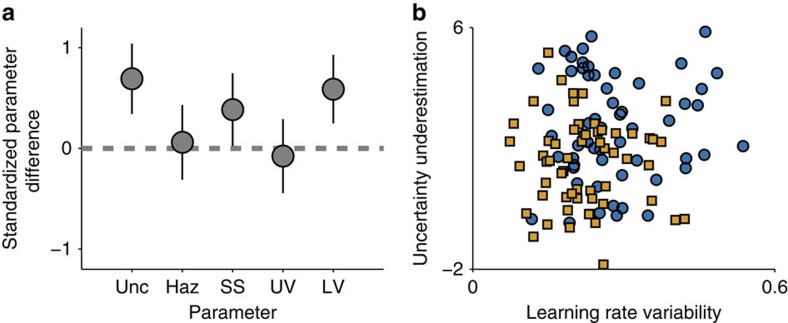
Age-related differences in uncertainty underestimation. Participant updating behaviour was fit with a flexible version of the normative model. (**a**) Mean and 95% confidence intervals on standardized differences (young–old) for maximum likelihood parameter estimates of: (1) uncertainty underestimation (*Unc*), (2) expectations about the hazard of change points (*Haz*), (3) sensitivity to surprising outcomes (*SS*), (4) update variability (*UV*), (5) learning rate variability (*LRV*). (**b**) Maximum likelihood estimates of uncertainty underestimation (ordinate) and learning rate variability (abscissa) for young (orange squares) and old (blue circles) participants.

Parameter estimates for young and old participants confirmed age differences in uncertainty along with additional differences (Fig. 6). Maximum likelihood estimates of the uncertainty–underestimation parameter tended to be positive, indicating that most participants were best fit by models that failed to represent normative levels of uncertainty (mean±s.e.m parameter estimates were 2.69±0.23 and 1.52±0.19 for old and young participants, respectively). Consistent with our descriptive results, this bias towards underestimating uncertainty was more pronounced in older participants (*t*=3.92, *n*=57 per group, *P*<0.001). In addition, we found age-related differences in surprise sensitivity, which was higher in older adults (mean±s.e.m. parameter estimates were 0.51±0.04 and 0.40±0.03 for young and old participants, respectively; *t*=2.11, *n*=57 per group *P*<0.05), and learning rate variability, which also was elevated in older adults as compared to younger adults (*t*=3.42, *n*=57 per group, *P*<0.001). In contrast, we found no evidence for differences in hazard rate across the two groups (*t*=0.21, *n*=57 per group, *P*>0.9).

One potential concern with using model-based parameter estimation to infer latent computational properties is that estimated parameters can be highly sensitive to the model in which they are embedded. In particular, the failure of a model to account for key sources of variability can lead to biased parameter estimates^33^. To examine the robustness of our modelling results, we constructed three additional models that progressively relax the assumptions about subjective perceptions of noise and in turn improved the ability of the model to account for behaviour. The first two models consider the possibility that subjective perceptions of noise are scaled (model 1) or scaled and offset (model 2) relative to the ground truth. The third model considers the possibility that noise is itself uncertain and predictive distributions are composed of a mixture of Gaussian distributions, each having a different width.

Consistent with previous work suggesting variability in perceptions of noise within and between subjects^18^, the most complex model containing within and between subject variability in noise estimates fit better than all simpler models, even after penalizing for additional parameters (Supplementary Fig. 4). The key advantage of this complex model appears to be that it effectively captures the shape of the relationship between learning rate and error magnitude (Supplementary Fig. 6). However, one disadvantage of this additional complexity is that it leads to parameter tradeoffs that decrease the recoverability of individual parameters, particularly the surprise sensitivity parameter (compare Supplementary Figs 5 and 7). Consistent with this lack of identifiability, surprise sensitivity did not differ across the age groups in any of these three models (all *P*>0.05). Nonetheless, the uncertainty underestimation and learning rate variability parameters were elevated for older relative to younger subjects in each of the three additional models (all *t*>3.4, all *P*<0.001 for UU, all *t*>3.1 all *P*<0.005 for LRV), suggesting that age differences in uncertainty underestimation and learning rate variability are robust to specific modelling choices.

Model estimates of uncertainty underestimation also offer a parsimonious description of age-related changes in learning. Subject-specific estimates of the uncertainty underestimation parameter were correlated with each of the four learning differences identified by our descriptive analysis (Pearson's *r*=−0.49, −0.20, 0.46 and −0.29 for learning rate, uncertainty, surprise and helicopter coefficients, respectively; all *P* values <0.05), suggesting that this metric can account for each of the features of the data that were identified using the descriptive model. Uncertainty underestimation fits were negatively correlated with fluid intelligence (*r*=−0.268, *P*<0.01), but not with working memory (*r*=−0.1287, *P*=0.17). Uncertainty underestimation also explained variance in age even after accounting for these potential covariates (nested F=5.92, *P*<0.05) and coefficients relating uncertainty underestimation to age were positive in a model that included these terms (mean±95% confidence interval: 1.59±1.28 years per unit uncertainty underestimation). Thus uncertainty underestimation offers a parsimonious explanation for age-related learning differences beyond the well-documented age differences in fluid abilities.

## Discussion

Age-related deficits in learning have previously been attributed to differences in the computation of prediction errors^3^,^5^,^6^. However, the data to support this hypothesis are somewhat contradictory and point to a more complicated scenario^34^. Here we examined one such scenario: deficits in learning result from differences in how older individuals assign influence to new information according to environmental statistics. We identified three plausible computational changes that could give rise to such a learning deficit: (1) insensitivity to surprise, (2) underestimation of uncertainty and (3) underestimation of the hazard rate of change points. To formalize these predictions, we simulated learning behaviour in a predictive inference task using a normative model and independently manipulated each of these factors. We then asked younger and older adults to perform the task and tested the model predictions empirically.

We found that older adults displayed a selective deficit in learning that is best described by reduced uncertainty. Older adults learned less from unsurprising outcomes than their younger counterparts, qualitatively matching the predictions from a low-uncertainty model of learning (compare Figs 2c and 4b). Moreover, older adults, like low-uncertainty learners, relied more heavily on surprise and less heavily on uncertainty in adjusting learning rates (compare Figs 4a,b and 5b,c). Consistent with the qualitative results of the regression analysis our quantitative model fitting suggests that age differences in learning can be explained by systematic underestimation of uncertainty by older adults (Fig. 6). Reduced uncertainty, as measured by parameter estimates from the flexible learning model, could explain differences in age even after accounting for age-related differences in working memory and reasoning abilities. These results support the overarching hypothesis that changes in learning across healthy aging result from changes in upstream computations necessary for determining how much to learn in a given situation. Furthermore, they suggest that age-related deficits in uncertainty-driven learning can be dissociated from a general decline in fluid abilities with age. Taken together, our data suggest that cognitive aging reduces subjective representations of uncertainty, which in turn diminishes learning under specific circumstances.

In addition, older adults in our study used more variable learning rates than their younger counterparts. This result is consistent with previous findings of age-related increases in behavioural variability^35^. Older adults also tended to have relatively enhanced sensitivity to surprise. However, we were unable to clearly dissociate this effect from their reduced uncertainty estimates. If future work could confirm such an advantage, surprise sensitivity might represent a compensatory mechanism that could be exploited in designing learning environments that are tailored to the needs of the elderly population.

While our results are the first to demonstrate a failure of older adults to recruit requisite levels of uncertainty in the service of learning, previous work has hinted that this might be the case. For example, recent work suggests that older adults show deficits in probabilistic compared with deterministic learning tasks^6^,^26^,^27^,^28^,^29^, in situations where feedback is ambiguous^30^,^31^ and during reversal learning^27^,^32^. Despite these deficits, older adults show similar competence to younger adults in decision tasks that explicitly describe risks, suggesting that the deficit is in learning from probabilistic cues, rather than failing to act appropriately according to learned values^9^,^11^,^12^. Our results suggest that these age-related impairments in learning can be attributed to a specific deficit in the ability to recruit requisite levels of uncertainty to appropriately guide learning.

Why do older adults fail to represent sufficient levels of uncertainty? One possibility is that representing appropriate levels of uncertainty requires a cognitive and/or biological resource that decays across healthy aging. One obvious candidate for such a resource is working memory capacity^36^. Working memory capacity declines as a function of healthy aging and has been identified as a potential source of age-related deficits in probabilistic learning^37^,^38^,^39^. While previous work has focused on the role of working memory for the storage and selective recall of action–outcome contingencies, another use for such a system might be to store and recall plausible hypotheses about latent task states^38^,^40^. In such a regime, decrements in working memory capacity would lead to fewer stored hypotheses, and in the extreme where only a single hypothesis is stored, a complete lack of uncertainty^41^. However, within our study we did not find strong evidence for a relationship between working memory capacity and uncertainty underestimation. Moreover, age-related deficits in uncertainty-driven learning persisted after controlling for age differences in working memory, arguing against this potential interpretation.

Another possibility is that older adults fail to represent sufficient levels of uncertainty because they have an aversion to uncertainty or the mental effort required to represent it. In descriptive lottery tasks younger and older adults are similarly averse to uncertainty about probabilities associated with possible gains suggesting that this explanation is unlikely to play a major role^42^. More generally, it is possible that older subjects are more averse to the expenditure of mental effort required to maintain an accurate uncertainty estimate and instead rely on a simpler learning strategy^43^. From our computational model it is not clear why representing low levels of uncertainty would be any easier than representing any other fixed level of uncertainty: the computational costs within our model are associated with updating, rather than maintaining, uncertainty estimates. One potential source of cognitive costs could be in the representation of the task model itself. There is some evidence that older adults tend to rely less on model-based strategies for learning^44^. In contrast, they tend to rely more on external cues to guide behaviour, which, in our task, could correspond to adopting a strategy like the ‘win-stay lose-shift' heuristic commonly used by older adults in choice tasks^45^,^46^. This general idea corresponds well to the double dissociation noted in our descriptive analysis of learning behaviour: Older subjects are more responsive to environmental learning cues (surprise) but much less responsive to internally generated ones (uncertainty).

The crucial factor limiting uncertainty representations in older adults could, and at some level must be, biological in nature. One candidate for such a limiting factor is norepinephrine, a neuromodulator thought to track uncertainty in changing environments^17^. There is some evidence that low-level arousal systems including brainstem noradrenergic nucleus locus coeruleus (LC) are modulated by uncertainty and may mediate its effects on learning. Pupil diameter, which is thought to reflect LC activity, is modulated by uncertainty in both learning and exploration tasks^16^,^47^,^48^. Activation of LC, and the corresponding increase in cortical norepinephrine, increases the signal-to-noise ratio in sensory neurons and may enhance learning by increasing the gain of cortical units representing newly arriving information^17^,^49^,^50^,^51^. A similar theory has already been proposed to explain age-related differences in learning^2^,^52^. Whereas previous accounts of the change in gain of information processing across age have focused on dopamine depletion as a possible explanation, there is also evidence that noradrenergic signalling may be dampened across healthy aging^53^,^54^,^55^, providing a potential link between our findings, the relationship between arousal and uncertainty, and the decreased cortical gain theory of aging.

Although it is tempting to link age-related changes in representing uncertainty to reduction of a single neurotransmitter, several alternative biological accounts exist. Functional imaging studies have highlighted uncertainty representations in prefrontal areas including the anterior prefrontal cortex (aPFC) and orbitofrontal cortex (OFC)^15^,^56^,^57^,^58^. There is substantial evidence for changes in the function and structure of the prefrontal cortex across healthy aging^59^. For example, prefrontal regions are substantially under-recruited in older versus younger adults when learning higher-order task structures^60^. Such under-recruitment could limit top-down activation of a cortical learning network during uncertain regimes^15^. Alternatively, diminished prefrontal recruitment during uncertainty could contribute directly to muted dopaminergic prediction error signalling, as prefrontal inputs play critical roles in shaping these signals^61^.

The existence of supporting evidence for both prefrontal and noradrenergic correlates of uncertainty representation highlights the need for future work combining computational methods with biological measurements and interventions that could unravel the underlying causal relationships between these factors and learning. Future studies should also investigate the extent to which biomarkers for uncertainty interact with the magnitude of reward-prediction error signals in the striatum. Taken in the context of previous work, our findings suggest that reward-prediction error signals in the striatum may be enhanced by neuromodulatory (LC) or cortical (aPFC) uncertainty signals. This would explain why prediction error signalling is diminished in older adults only under conditions of uncertainty, as these are the only conditions where reduced uncertainty representations would come into play^3^,^5^,^6^,^7^,^8^,^58^. While this mechanism provides a parsimonious explanation for previous findings, alternative accounts involving uncertainty representations computed locally in the striatum or the ventral tegmental areas are also plausible and should be tested through age comparisons of uncertainty modulations in the BOLD signal^62^,^63^.

Our results may also have implications beyond healthy aging for the understanding of a number of mental disorders for which learning deficits are a hallmark. In some cases, such deficits may not reflect an inability to learn but rather specific deficits in computational processes that govern how much to learn from new information. In addition to our results regarding aging, there has been recent support for this idea with respect to the effects of trait anxiety on learning^64^, but to date it is unknown whether learning abnormalities in conditions such as attention deficit hyperactivity disorder (ADHD), autism or schizophrenia are also driven by higher-order computational factors. Our task and modelling framework provide a means to address these issues^65^.

To summarize, in the current study we apply a normative model of predictive inference to simulate possible effects of aging on three factors that are thought to govern learning: uncertainty, surprise and hazard rate. Using behavioural data and quantitative model fitting we show that learning deficits in older adults are best characterized by an underestimation of uncertainty rather than a generic reduction of learning. This finding provides a parsimonious mechanistic explanation for age-related impairments in learning across a variety of tasks. Furthermore, it highlights specific cortical and subcortical regions involved in representing uncertainty as candidates for mediating age-related learning deficits. We hope that this work facilitates future studies aiming to understand the neural underpinnings of limited uncertainty representation and age-related changes thereof.

## Methods

### Participants

59 younger and 63 older adults took part in the study. Target sample size was pre-determined according to age effect sizes in previous studies of learning and decision-making. Six older and two younger adults were excluded because of insufficient data (<300 trials of predictive inference task completed). Thus, the effective sample consisted of 57 younger adults (mean age: 24.5 years, age range: 20–30 years, 29 female) and 57 older adults (mean age: 69.2 years, age range: 56–80 years, 26 female). Participants gave written informed consent. The Institutional Review Board of the Max Planck Institute for Human Development approved the study. In addition to the experimental task, participants completed a biographical and a personality questionnaire and several psychometric tests: (1) Identical pictures test; (2) Raven's Progressive matrices^66^; (3), Spot-a-Word test; and (4) the Operations span task (OSPAN)^67^. As shown in Table 1 older adults had lower scores on the Identical pictures test, Raven's matrices and the OSPAN task than younger adults (*P* values<0.001, *η*_G_^2^s>0.21). In contrast, older adults obtained higher scores than younger adults on the Spot-a-Word test (*P*<0.001, *η*_G_^2^=0.20). Consistent with previous findings from larger population-based samples, these results suggest age-related reductions in fluid intelligence and age-related improvements in crystallized intelligence^68^.

### Procedure

Participants performed two sessions, which were separated by a minimum of 1 week and a maximum of 3 weeks. In the first session, participants completed a biographical questionnaire, the BIS/BAS personality questionnaire, Raven's progressive matrices^66^ as well as a two-state Markov decision task^69^, data of which are presented in ref. 44. In the second session, participants performed the predictive inference (Helicopter) task^15^, the OSPAN task, the Spot-a-Word and the Identical pictures test as well as a version of the two-state Markov task.

### Predictive inference task

Participants completed two blocks (200 trials each) of a computerized predictive inference task programmed in Matlab (The MathWorks, Natick, MA) using MGL (http://justingardner.net/mgl) and snowDots (http://code.google.com/p/snow-dots) extensions. The predictive inference task required inferring the mean of a noisy variable that underwent occasional change points^15^. The problem was embedded in a cover story involving a virtual helicopter (mean) that moved occasionally (change points) and dropped a bag from the sky on each trial (noisy variable).

On each trial the participant moved a bucket to the most likely position of the helicopter using a keyboard (Fig. 1a). After the bucket position was confirmed through a key press, the participant observed a bag fall from the top of the screen followed by an explosion that revealed the contents of the bag (200 gold coins or silver rocks; randomized across trials) and the extent to which those contents were collected in the bucket (ranging from 0–200 depending on the distance between the bucket and the bag). Gold tokens (but not rocks) collected in the bucket were translated into incentive payments at the end of the task. The horizontal position of the bag was denoted with a grey tick mark on the screen and the distance between the bag and bucket (prediction error) denoted by a red line. These markings served to eliminate working memory requirements and allowed subjects access to all relevant information in choosing how much to adjust the bucket position for the subsequent trial.

The horizontal position of each bag (represented on a numerical scale from 0 to 300 for convenience) was drawn from a normal distribution with a mean corresponding to the position of a virtual helicopter hovering in the sky and a s.d. that was manipulated blockwise (10 or 25; counterbalanced for order). On most trials the helicopter would remain stationary, but on a small fraction of trials (ground truth hazard rate; 1/10) it would relocate to a new screen position. On the vast majority of trials the helicopter was ‘hidden' by clouds. Occasionally, the helicopter was revealed visually (catch trials; 1/10). In principle, the visible helicopter could provide perfect information about the mean of the distribution, but in practice the centre of the helicopter was not obvious due to asymmetry in the cartoon helicopter image and the vertical distance between this image and that of the bucket (Supplementary Fig. 1). Participants were instructed to infer the location of the helicopter based on previous observations (bag and helicopter positions) and to place the bucket directly underneath it.

### Training

Before completing two blocks of the predictive inference task participants went through a series of training tasks that slowly built the complex task from simpler elements. As in the experimental session, every training task consisted of a low and high standard deviation (noise) block (counterbalanced for order). In the first training task the helicopter was completely visible and thus bag locations were not necessary to guide behaviour. To ensure that participants understood that the helicopter is the best outcome predictor we used a response criterion that required participants to put their bucket ten times exactly underneath the visible helicopter. Each noise block stopped after either the criterion was reached or after a maximum of 80 trials. In the second training task with two 50-trial runs clouds covered the helicopter and occasionally disappeared during catch trials. This version of the task was the same as the experimental task except that participants would not earn money for their collected coins. Overall performance, in terms of coins collected, did not differ across age groups (Supplementary Fig. 2).

### Computational modelling

To dissociate surprise-driven updating from uncertainty-driven updating we extended an existing normative model for learning in a dynamic environment that has been described in detail previously^15^,^16^,^18^. In brief, this model approximates optimal inference by tracking two factors that should drive learning: change-point probability (the probability with which a change in the helicopter location occurs) and uncertainty (the reliability with which an outcome reflects the true location of the helicopter). Here we extend this model in four ways. First, we develop a new method for estimating change-point probability and uncertainty that captures subjective differences in experienced surprise. Second, we extend the generative framework and corresponding inference equations of the model to incorporate catch trials. Third, we extend the normative model to allow for specific deviations from normativity including surprise insensitivity, incorrect hazard rate assumptions, and uncertainty underestimation. Finally, we extend the model to consider more complex models of behaviour that allow for subjective differences in the representation of noise.

The first extension of the previously described computational methods allowed for subjective estimates change-point probability and uncertainty. Previous studies have run the normative model over trial outcomes to get trial-by-trial estimates of these quantities^16^; however, one issue with this approach is that since participant and model predictions do not always perfectly match, an outcome that constitutes a small and unsurprising error for the model might actually be a large and rather surprising one for the participant. To avoid this potential problem we obtained subjective measures of change-point probability and uncertainty by running the normative model across the prediction errors experienced by participants, rather than the outcomes that generated them. Model variables were computed recursively by first determining the uncertainty about the current helicopter location according to the relative uncertainty, change-point probability and prediction error from the previous trial:


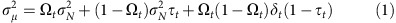


Where 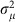 is the variance on the predictive distribution over possible helicopter locations, 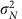 is the variance on the distribution over bag locations (noise), *Ω*_*t*_ is the probability of a change point on the previous trial (that is, the probability with which the helicopter has relocated between trials), *τ*_*t*_ is the relative uncertainty from the previous trial and *δ*_*t*_ is the prediction error from the previous trial. Relative uncertainty was computed by expressing uncertainty about the helicopter location as a fraction of total uncertainty about the location of the next bag:


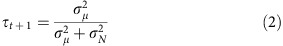


Where *τ*_*t*+1_ is the relative uncertainty for trial *t+1*. This relative uncertainty estimate, along with the variance on the bag distribution (noise; 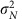) was used to calibrate the change-point probability associated with each new prediction error:


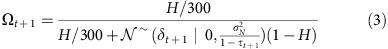


Where *H* is the hazard of a change point (0.1) and *δ*_*t*+1_ is the new prediction error. Subjective estimates of change point probability and relative uncertainty were computed by evaluating equations 1 and 2 according to the trial-by-trial prediction errors made by each individual subject.

The second extension of the model was necessary to account for additional information provided on catch trials in which the helicopter is visible to participants. To maintain the deterministic nature of the model but also account for perceptual ambiguity associated with the helicopter image we treat the visible helicopter as a cue indicating a Gaussian likelihood function centred on the ground truth (mean of the bag distribution). We allow the variance of the Gaussian to be adjusted to account for behaviours ranging from completely trusting the helicopter information to completely ignoring it. Combining this additional cue with the information provided by the bag itself led to the following additional equations that were implemented at the end of each helicopter visible trial to update position estimates:


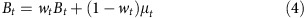



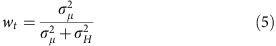


Where *B*_*t*_ is the belief of the model about the true mean of the distribution and *w*_*t*_ reflects the weight of the current belief in a weighted mixture of the current belief and the true mean (μ) as indicated by the helicopter. *w*_*t*_ is determined according to the relative variances on the current predictive (σ_*μ*_) and helicopter centred likelihood distributions (σ_*H*_).

In addition, the following equations were implemented to reduce the relative uncertainty estimates on trials where the helicopter was observable:


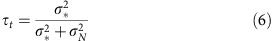


Where 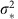 is the variance on the predictive distribution over possible helicopter locations after correcting for additional information provided by the visible helicopter:


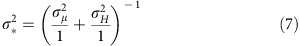


Where 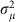 and 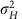 are the variances associated with the internal prediction and the perceptual information provided by the visible helicopter, respectively.

The third extension of the normative model served to allow for specific deviations from optimal behaviour. We simulated behaviour from four versions of the normative model: (1) a version using the update equations described previously^15^,^16^ with the modifications described above, (2) a model with diminished sensitivity to surprise created by raising the change point likelihood to a power between 0 and 1 (0.2 for figures) as described previously^18^, (3) a low hazard rate model expecting change points to be rare (*H* was set to 0.001) and (4) an uncertainty underestimation model in which uncertainty was reduced after each observed bag drop by dividing the estimated variance on the predictive distribution over possible helicopter locations (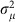) by a constant on each trial (10 for simulations).

Flexible versions of the normative model were fit directly to behaviour and used to infer maximum likelihood estimates of (1) hazard rate, (2) surprise sensitivity and (3) uncertainty underestimation, which were then use to identify age-related differences in these computational factors. For the purposes of model fitting, participant updates were defined to be sampled from a normal distribution with a mean equal to the model predicted update and a s.d. that was a linear function of the absolute prediction error magnitude. The intercept and slope of this linear function were fit as free parameters and can be thought of as variability in the motor update and learning rate selection processes respectively. Thus, the minimally complex model contained five free parameters, three of which were related to learning and two of which were related to response variability. This model fit better than several more constrained ones in which parameters were fixed to their normative values (Supplementary Fig. 4).

In addition, more complex models were constructed that considered potential sources of variability related to the perception of noise. These complex models included all of the basic variables as well as one or more of the following free parameters: (1) a multiplicative scaling term to allow for scaled perceptions of noise, (2) an additive offset term allowing for subjective biases in overall levels of noise perception and (3) a noise variability term allowing for individual subjects to represent a distribution across possible noise values. Since there were only two noise conditions, including additive and multiplicative scale factors amounted to allowing the noise for each block type to be fit as a free parameter. Within the model that accounted for noise variability, the likelihood of observations was not drawn from a single normal distribution (as described in equation 3), but instead from a weighted mixture of normal distributions, where each component of the mixture had a mean of zero and a s.d. equal to a scaled version of the total uncertainty. Scale values were represented as uniformly spaced points on a grid (ranged 0.1–100) with associated probabilities drawn from an inverse gamma distribution. The shape term of the gamma distribution was fit as a free parameter and can be thought of as conveying the amount of evidence for the expected noise distribution, with lower values indicating more uncertainty over possible noise values.

All models were fit using a constrained search algorithm (fmincon in Matlab) that maximized the total log posterior probability of participant updates given participant prediction errors and parameter estimates. Weak priors favouring normative learning parameters were used to regularize parameter estimates. Uncertainty underestimation estimates were positively skewed and thus reported and analysed in log units. All model-fitting code will be made available on request.

### Data analysis

Participant bucket placements and trial outcomes were used to compute trial-by-trial prediction errors (*δ*):


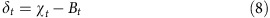


where *χ*_*t*_ and *B*_*t*_ are the locations of the dropped bag and placed bucket on trial *t*, respectively. The corresponding updates made by the participant in response to each prediction error were computed as:


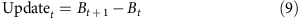


The first and last trials of each block were omitted from further analysis, as updates on these trials were likely to be influenced by block changes. Trials where the prediction error equalled zero were also omitted, as they provide no information about error-driven learning. In addition trials where bucket placement fell more than 15 screen units away from any possible delta rule update towards the previous bag or helicopter position were omitted, as they were considered to be governed by a process other than error-driven learning. 1.1% of trials were removed in this way.

Trial-by-trial updates were analysed with a regression model that included trial-by-trial prediction errors to account for overall learning rate, as well as the interaction of prediction error with five mean-centred factors: (1) surprise (change-point probability as computed above), (2) uncertainty (relative uncertainty as computed above), (3) noise (s.d. of bag distribution), (4) trial value (gold versus rocks) and (5) helicopter visibility (binary variable describing whether helicopter cue was provided). To allow for updates towards the visible helicopter on catch trials, the model also included the interaction between the true mean of the distribution and the helicopter visibility variable. An additional nuisance term was also included to account for a slight bias in bucket placements towards the centre of the screen. One potential shortcoming of this regression model is that the residuals are heteroscedastic; specifically, absolute residuals are larger on trials where participants made larger absolute prediction errors. To account for this, we used an initialization regression for each participant and pooled the residuals to compute the variance over residuals across sliding windows of absolute prediction error magnitude. These variance estimates were used to weight the errors in a weighted regression equation that also included a ridge penalty to regularize coefficient estimates:


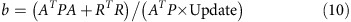


where *A* is the explanatory matrix, *P* is the inverse variance matrix, and *R* is a regularization matrix constructed with the ridge parameter equal to 0.1.

To identify specific learning differences predicted from the normative model (Fig. 2), we applied the penalized weighted regression model to data that were binned in sliding windows according to the size of the absolute prediction error made by the participant divided by standard deviation of the bag distribution, which served as a proxy for surprise (Fig. 3a). Each bin contained 10% of the total data and successive bins had lower and upper bounds that were incremented by a single percentile resulting in 90 total bins.

Regression coefficients were smoothed across bins and t-tests were used to identify ‘clusters' of contiguous bins for which the p-value was smaller than 0.05. This procedure was repeated for three separate tests to reject the null hypotheses that (1) coefficients from older participants are different than zero, (2) coefficients from younger participants are different from zero and (3) coefficients from younger participants are different from those from the older participants. For each cluster, we computed cluster mass as the size of the cluster (number of bins) times the average absolute *t*-statistic within that cluster. For each test statistic a null distribution over cluster mass was generated by creating 10,000 permutations of the data (using sign-flipping for single group tests and label-flipping for the group comparison). Cluster corrected permutation tests were conducted by comparing the observed cluster mass for each test statistic against the null distribution created through these permutations. See Supplementary Fig. 3 for estimates of parameters that did not differ across the age groups.

Single participant coefficients were extracted for each coefficient that was significantly different across age groups according to a leave-one-subject-out (LOSO) procedure: Coefficients for each participant were extracted from the error bin that corresponded to the maximum absolute *t*-statistic from a between groups *t*-test across all bins for all other participants. These LOSO coefficient estimates were included as explanatory variables in a regression on participant age. Specifically, we created four distinct explanatory models containing: (1) only an intercept term, (2) LOSO coefficients and an intercept, (3) LOSO coefficients, Raven's scores, OSPAN scores and an intercept. Nested F-tests were used to compare the fits of these different models while accounting for differences in complexity.

## Additional information

**How to cite this article:** Nassar, M. R. *et al*. Age differences in learning emerge from an insufficient representation of uncertainty in older adults. *Nat. Commun.* 7:11609 doi: 10.1038/ncomms11609 (2016).

## Supplementary Material

Supplementary Information Supplementary Figures 1-7

## Figures and Tables

**Table 1:** Psychometric profiles of younger and older adults.

**Psychometric**	**Younger adults (*****N*****=57, mean age: 24.5, range: 20–30)**	**Older adults (*****N***=**57, mean age: 69.1, range: 56–80)**
**measure**	**(mean, s.e.m.)**	**(mean, s.e.m.)**
Raven (raw score)	12.3 (0.4)	6.3 (0.4)
Ospan total score	54.3 (1.7)	39.9 (2.2)
Processing speed	31.1 (0.6)	21.2 (0.5)
Spot-a-Word	19.9 (0.8)	25.7 (0.7)
