# Supplementary material for: Age differences in learning emerge from an insufficient representation of uncertainty in older adults
Source: Nat Commun. 2016 Jun 10;7:11609. doi: 10.1038/ncomms11609 (PMC4906358; doi:10.1038/ncomms11609)
Supplement: Supplementary Information — Supplementary Figures 1-7 [file ncomms11609-s1.pdf]

Supplementary Information

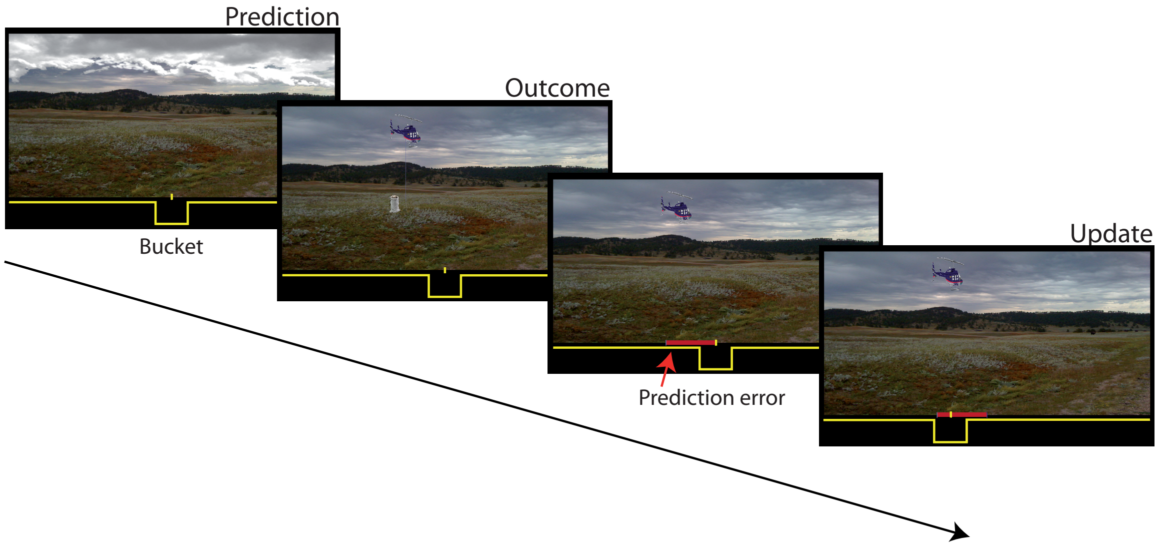

Supplementary figure 1: Helicopter visible catch trials. On 10% of trials the helicopter would become visible (clouds moved off-screen) before the bag was dropped. While the bag dropped, a gray line extended from the helicopter to the ground marking the center of the helicopter. After the bag reached the ground it exploded revealing either "coins" or "rocks" and the gray line disappeared. Some of the contents would fall in the bucket and count toward incentive payments. The participant would then be allowed to update the bucket position in full view of the helicopter (but not the gray line marking its center).

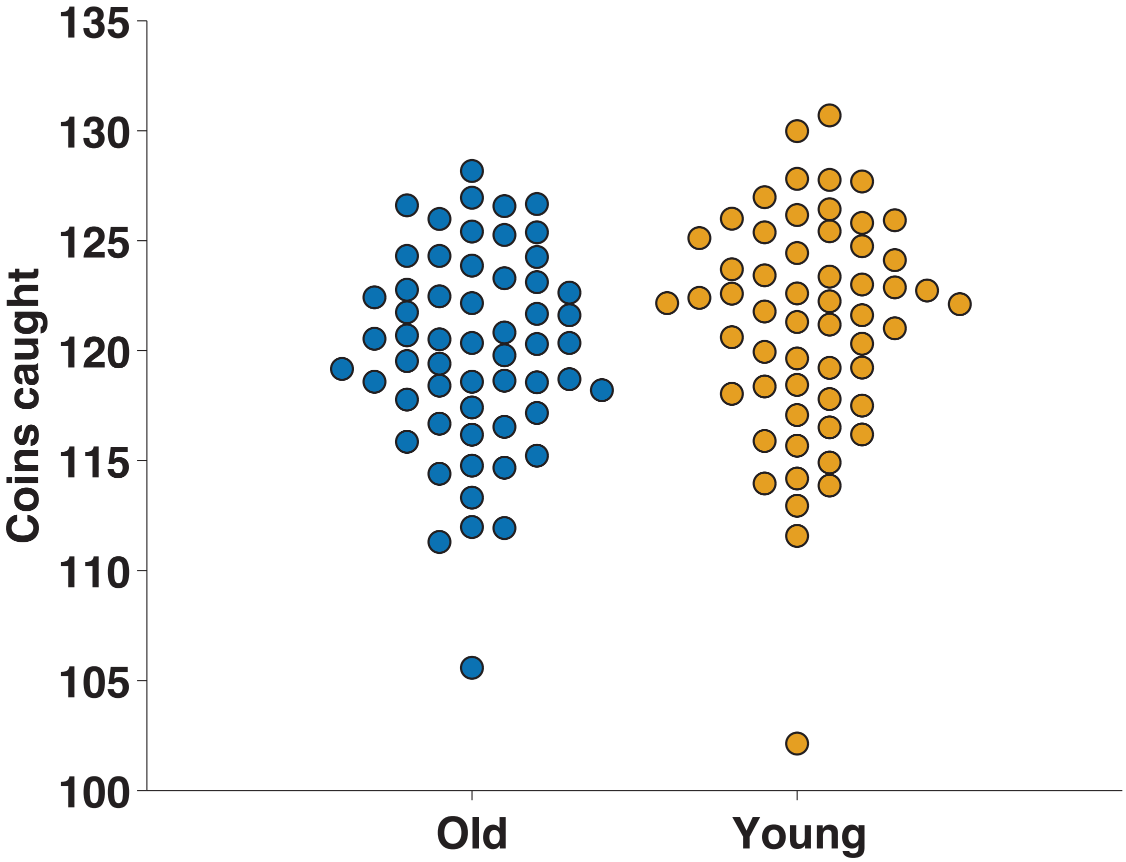

Supplementary figure 2: Participant performance. Mean performance on non-change-point trials (ordinate) is plotted separately for younger (yellow) and older (blue) participants separately (abscissa values are jittered to display data for all participants). Younger subjects collected more coins on average, but the difference between groups was small relative to within group variability (t-test for difference between young and old subjects: $t=-1.71$, $n = 57$ per group, $p=0.09$).

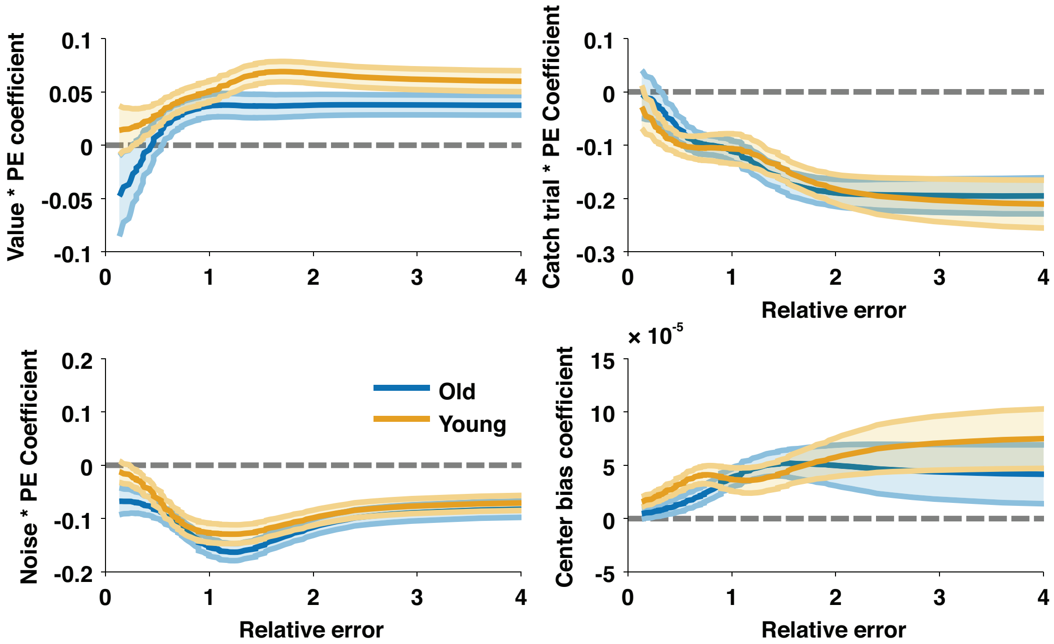

Supplementary figure 3: Regression coefficients for additional explanatory variables. Coefficients were computed from weighted ridge regression on participant updates binned according to relative error magnitude as described in results and methods. All coefficients took a value that differed significantly from zero after correcting for multiple comparisons. **A.** The interaction between value and prediction error revealed positive coefficients for both age groups, suggesting that both groups adjusted learning according to outcome value, even though doing so is inconsistent with normative learning in this particular task. **B.** Both older and younger participants tended to use outcome information less on catch trials (during which the helicopter was visible). **C.** Both groups updated less in response to a given relative error when in the high noise condition. **D.** Both groups tended to bias updates away from the edges of the computer display.

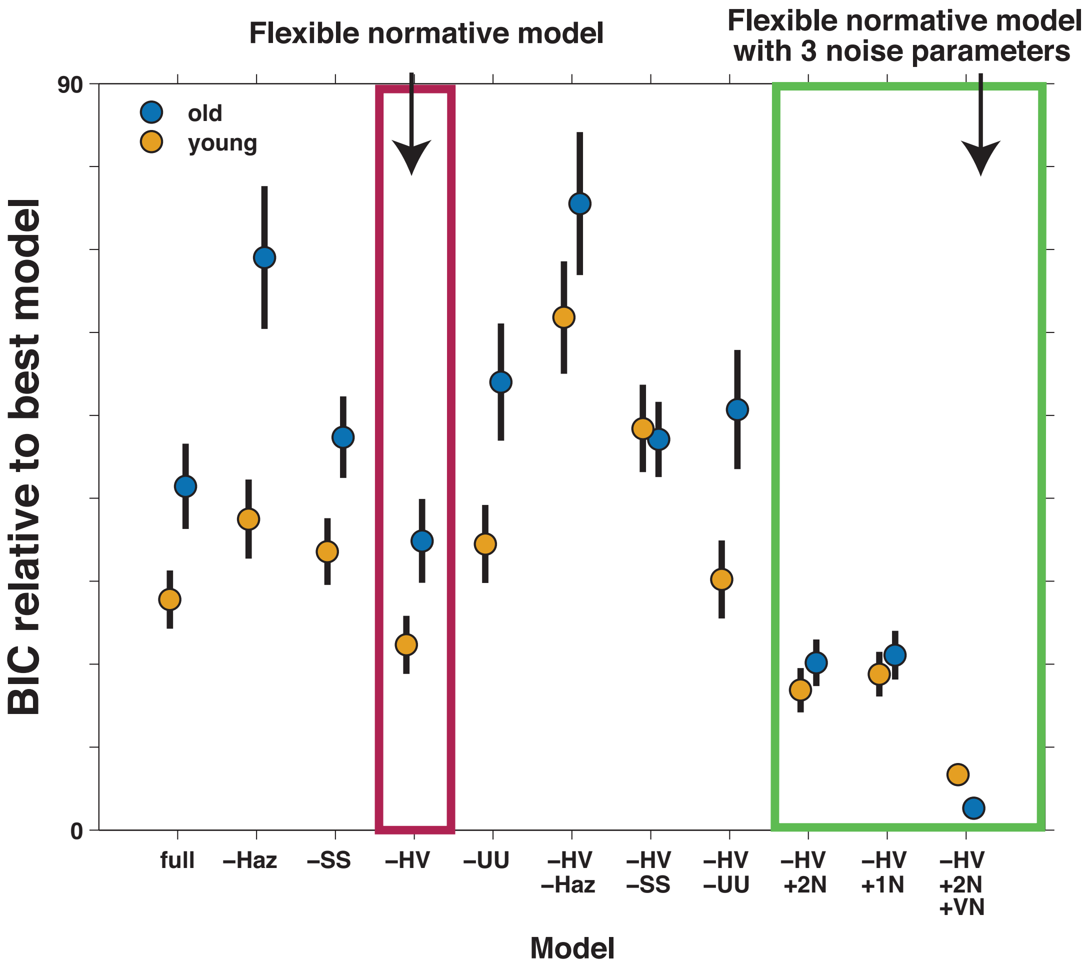

Supplementary figure 4: Model selection for flexible normative model. Bayesian Information Criterion (BIC) was computed per participant for several model parameterizations including a full model where hazard rate (haz), surprise sensitivity (SS), uncertainty underestimation (UU), and the uncertainty associated with the visible helicopter cue (HV) were all fit as free parameters. In addition we tested several models that fixed one or more of these factors to their normative values (where missing terms are designated with a minus sign). Finally, we also considered models that relax assumptions about subjective perceptions of the noise distribution. These models allowed for noise estimates that were scaled by a multiplicative factor (+1N) or scaled and offset (+2N) allowing subjective noise to be fit freely for each of the 2 noise conditions. We also considered an extension of the model that allowed for variable noise estimates within a block of trials (+VN; see

methods for details). For each participant a relative BIC score was computed by subtracting the lowest BIC for any model from all models. Of the basic models that made strong assumptions about noise perceptions, relative BIC scores were lowest for the model that left hazard rate, surprise sensitivity and uncertainty underestimation as free parameters but fixed the uncertainty associated with the visible helicopter cue to zero (red rectangle). Adding additional free parameters to relax assumptions about subjective noise perceptions improved fits (green rectangle) but led to problems with parameter recovery (compare supplementary figure 5 to supplementary figure 7).

Nassar et al.
Supplementary Figure 5

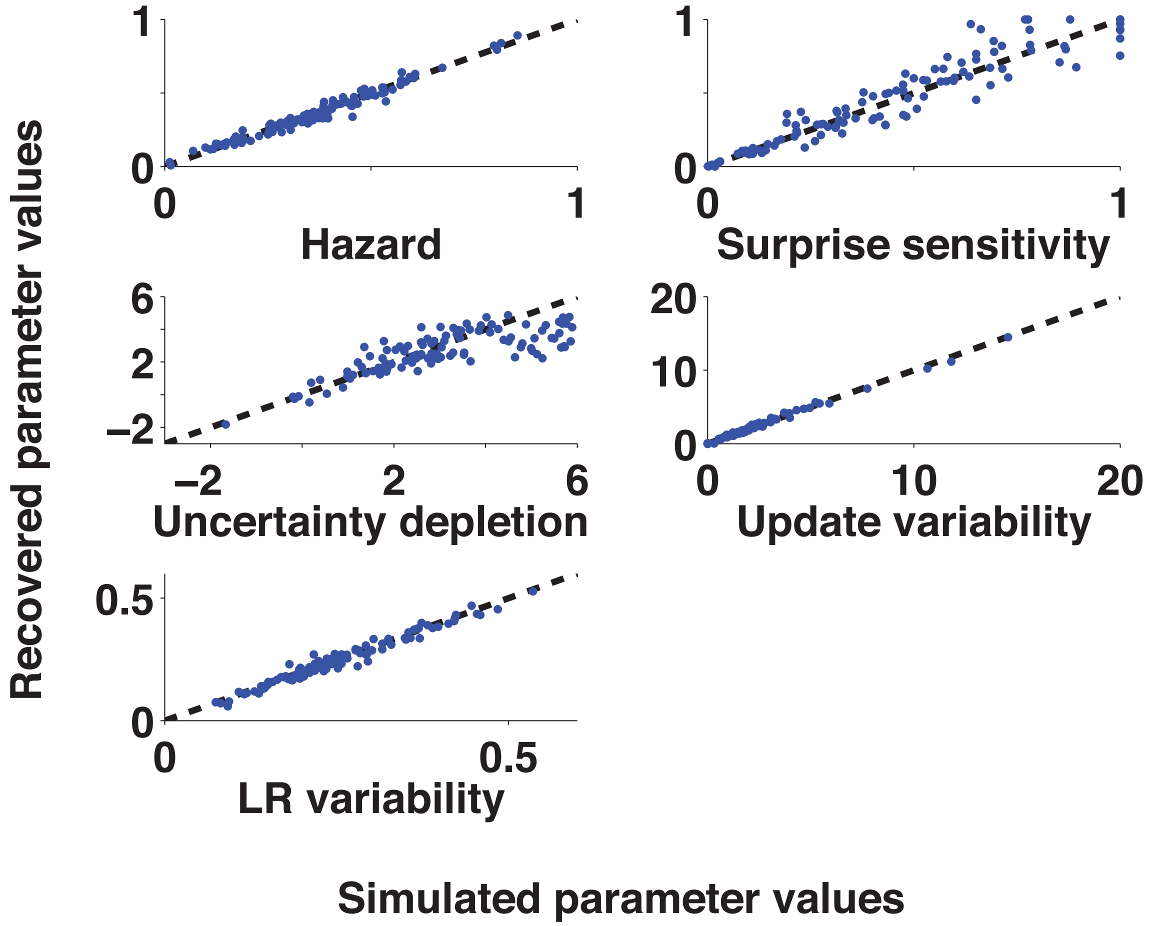

Supplementary figure 5: Simulation and recovery of model parameters in basic model.
Simulated (abscissa) and inferred values (ordinate) for each model parameter (hazard, surprise sensitivity, uncertainty underestimation, update variability and LR variability) for each simulated dataset (blue points). The fitting procedure used for parameter recovery exactly matched that used to infer parameters from subject data. Correlation between simulated and inferred parameter values was greater than 0.8 (pearson's rho) for each of the 5 parameters.

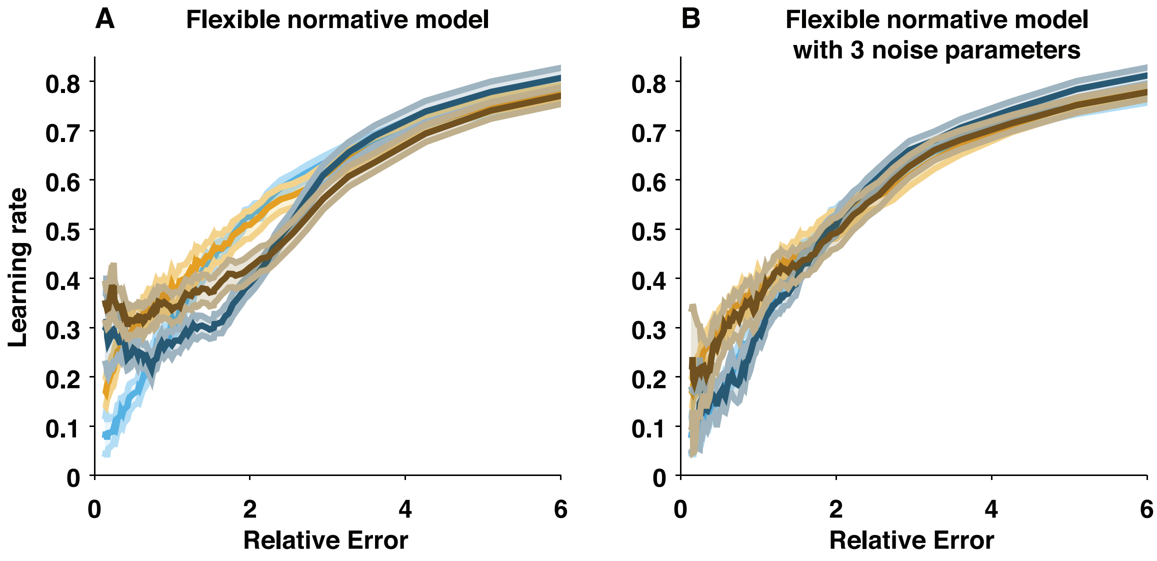

Supplementary figure 6: Posterior predictive check of basic and extended model fits.
A&B. Mean/SEM regression coefficients (line/shading) describing the effect of prediction errors on updates are plotted separately for older (light blue) and younger (light orange) participants across all sliding window bins of unsigned relative error along with the same analysis applied to data simulated from best fitting basic model (panel **A**; dark colors) or best fitting extended model (panel **B**; dark colors). Consistent with basic model selection criteria (supplementary figure 4), the extended model including free parameters to allow for individual differences in noise perceptions better matches the data. In particular, relaxing assumptions about noise appears to allow the shape of the learning curve to better match that of the subjects.

Nassar et al.
Supplementary Figure 7

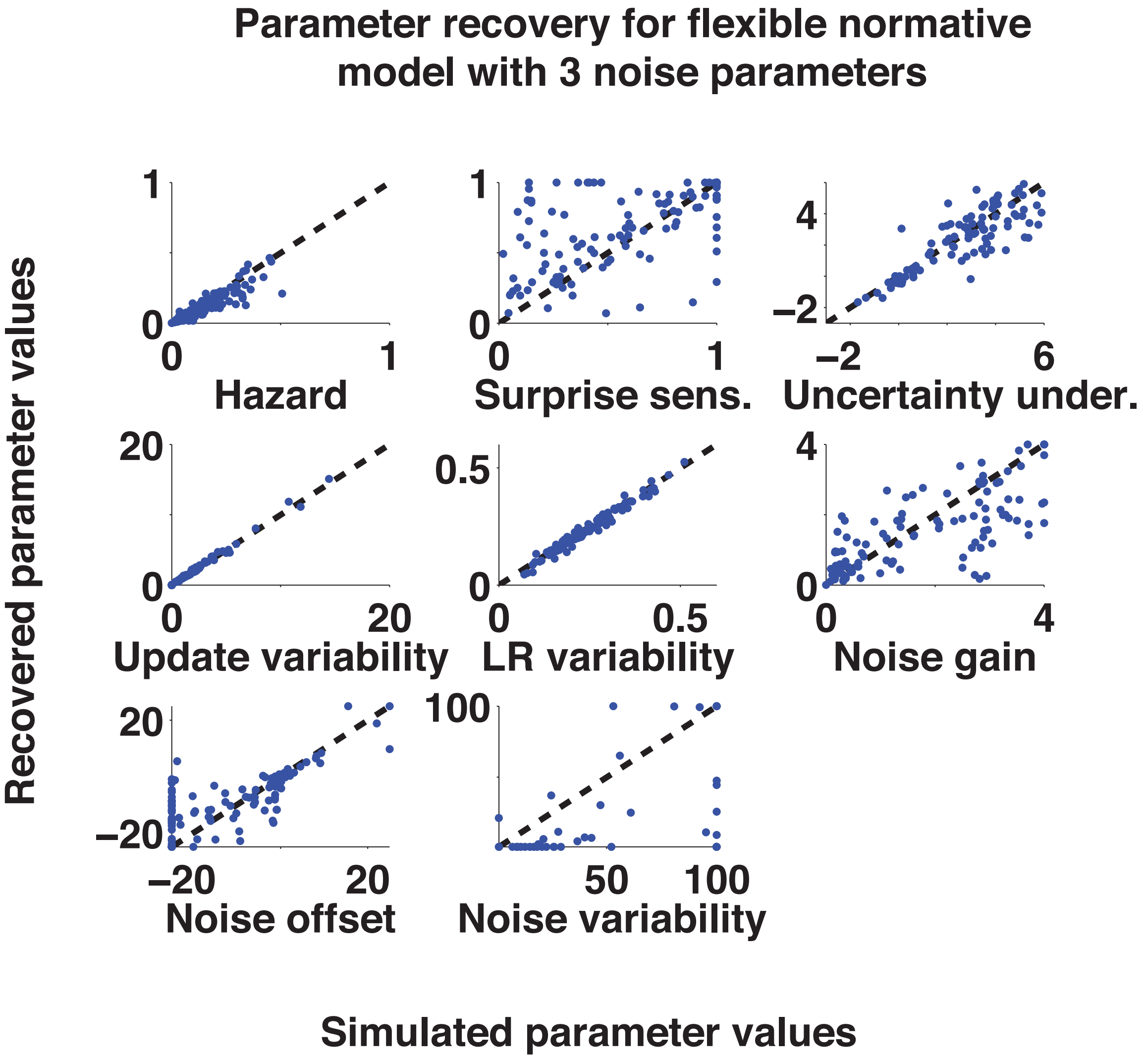

Supplementary figure 7: Simulation and recovery of model parameters in extended model.

Simulated (abscissa) and inferred values (ordinate) for each model parameter (hazard, surprise sensitivity, uncertainty underestimation, update variability, LR variability, noise gain, noise offset, noise variability) for each simulated dataset (blue points). The fitting procedure used for parameter recovery exactly matched that used to infer parameters from subject data. Despite the improved fit provided by this extended model, parameter values were not consistently recovered, particularly for the surprise sensitivity and noise parameters.
